# Bacterial communities associated with ambrosia beetles: current knowledge and existing gaps

**DOI:** 10.3389/fmicb.2025.1569105

**Published:** 2025-04-09

**Authors:** Juan Carlos Cambronero-Heinrichs, Peter H. W. Biedermann, Laura Besana, Andrea Battisti, Davide Rassati

**Affiliations:** ^1^Department of Agronomy, Food, Natural resources, Animals and Environment (DAFNAE), University of Padova, Legnaro, Italy; ^2^Centro Nacional de Innovaciones Biotecnológicas (CENIBiot), CeNAT-CONARE, San José, Costa Rica; ^3^Chair for Forest Entomology and Protection, University of Freiburg, Stegen, Germany

**Keywords:** ambrosia beetles, symbiosis, mutualism, bacteria, bacterial communities

## Abstract

Ambrosia beetles (Curculionidae: Scolytinae and Platypodinae) are wood-boring insects studied as examples of fungus-insect symbiosis and for their success as invasive species. While most research on their microbiota has focused on fungal associates, their bacterial communities remain largely understudied. In this review, we synthesize current knowledge on the bacterial microbiota of ambrosia beetles, identify critical gaps in the field, and provide recommendations for future research. To date, eight metabarcoding studies have explored bacterial communities in ambrosia beetles, analyzing a total of 13 species, mostly within the tribe Xyleborini (Scolytinae). These studies have examined the presence of bacteria in ambrosia beetle mycetangia, organs specialized for transporting fungal symbionts, as well as bacterial diversity in fungal gardens and whole beetles, across different life stages, and under varying environmental conditions. In general, bacterial communities appear to be highly specific to the beetle species, and differ between the beetles and their fungal gardens. Most studies employed 16S rRNA gene metabarcoding, and the optimal primer combination for characterizing bacterial communities in environmental samples is 515F/806RB (V4). Various methods for collecting beetles have been used, such as ethanol-baited traps, direct collection from galleries, logs kept in emergence cages, and rearing, but which of them to select when planning a study depends on the specific aim. A significant knowledge gap remains regarding the functional roles of dominant bacterial taxa, as metabarcoding studies often assume that these roles are similar to those played in other beetle species, such as bark beetles. More studies should be conducted to test hypotheses regarding the various factors influencing microbial composition and function, and advanced molecular techniques, including (meta-) genome and transcriptome sequencing, which have been employed in only a limited number of studies, could offer great potential to help bridging this knowledge gap.

## 1 Introduction

Ambrosia beetles in the subfamilies Scolytinae and Platypodinae (Curculionidae) are wood-boring insects that typically excavate tunnels into the xylem of their host trees (Kirkendall et al., 2015). Their defining characteristic is the obligate mutualistic and nutritional association with fungi, which they cultivate in their tunnels inside living or freshly dead trees (Dzurenko and Hulcr, 2022; Hulcr and Skelton, 2023). Depending on the species, fungi either comprise the majority (xylomicetophagous species) or entirety of the ambrosia beetle diet (mycetophagous species) (Roeper, 1995). This obligate fungal mutualism independently evolved at least 16 times in ambrosia beetles (Johnson et al., 2018). Spores of their nutritional ambrosia fungi are carried in specialized organs called mycetangia, which shape and position on the beetle body change depending on the clade of fungi the beetles are associated with (Li et al., 2018; Mayers et al., 2022). Glands within mycetangia are only active during beetle dispersal and produce yet unknown substances that select for almost pure ambrosia fungus content (Mayers et al., 2022). When beetles are inside the nests, the mycetangia are instead not activated and contain a more or less complex mixture of fungal species (Mayers et al., 2022).

The interest toward ambrosia beetles has exponentially raised over the last decades following the numerous invasions favored by increasing trade and climate change (Lantschner et al., 2020; Pureswaran et al., 2022). Despite most of these introductions did not cause evident impacts, a few ambrosia beetle species have become main pests in the areas of introduction (Hulcr and Stelinski, 2017). For instance, the redbay ambrosia beetle *Xyleborus glabratus* and its pathogenic ambrosia fungus symbiont, *Harringtonia lauricola*, devastated plant species in the family Lauraceae in North America (Hughes et al., 2017), with cascade effects on associated arthropod communities (Riggins et al., 2019). The polyphagous shot hole borer *Euwallacea fornicatus* caused major damages to urban trees in California and other invaded areas (e.g., Coleman et al., 2019), and threatened urban trees and avocado industry in Israel (Mendel et al., 2017) as well as tropical greenhouses in Europe (Schuler et al., 2023). *Xylosandrus crassiusculus* and *Xylosandrus germanus* are pests in ornamental tree nurseries and orchards in North America (Ranger et al., 2016; Gugliuzzo et al., 2021). These invasions inevitably led several researchers around the globe to invest efforts and resources to learn more about ambrosia beetle biology and ecology as starting point to develop possible management strategies (Hulcr and Stelinski, 2017; Hulcr et al., 2020).

Among the most studied aspects of ambrosia beetles is the symbiosis with nutritional and non-nutritional fungi (Hulcr and Skelton, 2023; Osborn et al., 2023). Communities of fungi present inside mycetangia (e.g., Kostovcik et al., 2015; Mayers et al., 2015; Joseph and Keyhani, 2021), on the beetle body (e.g., Bateman et al., 2016; Malacrinò et al., 2017; Skelton et al., 2018; Rassati et al., 2019; Morales-Rodríguez et al., 2021), and inside tunnels (e.g., Diehl et al., 2023; Mahony et al., 2024) have been described in many species, clearly showing that filamentous fungi and yeasts (Davis, 2015) are constant components of ambrosia beetle mycobiota along with the nutritional fungal symbionts. On the contrary, the number of studies which investigated the bacterial communities associated to ambrosia beetles is still low. Based on data retrieved from PubMed, only eight studies have been conducted, representing approximately 0.3% of all studies on insect-associated bacterial communities and less than 0.2% of those investigating insect microbiota (including all microbe types). This is clearly an important knowledge gap as bacteria are widespread associates of ambrosia beetles (Hulcr et al., 2012; Ibarra-Juarez et al., 2020; Diehl et al., 2022; Calleros-González et al., 2024) and some of them might play beneficial roles, potentially acting as third players in beetle-fungus mutualisms. For instance, several molds coexist with nutritional fungi within tunnels (Diehl et al., 2023), and bacteria may act as defensive symbionts that inhibit these competitors (Grubbs et al., 2011; Cambronero-Heinrichs et al., 2023). The same mechanism has been in fact observed in other insect-fungus farming systems (Currie et al., 1999). Bacteria have been also linked to nutritional benefits, such as lignocellulosic activity and supplementation of essential amino acids (Cambronero-Heinrichs et al., 2023). Such nutritional functions are also well documented for the bacterial communities of other insects that rely on plant material for fungiculture (e.g., termites; Murphy et al., 2024). However, prior to this study, no publication summarized the knowledge on bacterial symbionts of ambrosia beetles, although there are a few reviews addressing the role of bacteria in the closely related bark beetles (Six, 2013; Hofstetter et al., 2015; García-Fraile, 2018; Saati-Santamaría et al., 2021).

The present review aims to: (i) summarize the existing knowledge on bacterial communities associated with ambrosia beetles; (ii) describe the sampling methods employed to obtain adult individuals for studying their associated bacterial community, as well as the molecular approaches used for analysis; and (iii) highlight the key knowledge gaps that future research should address. To achieve these aims, we reviewed the key findings reported in studies that used metabarcoding to characterize the bacterial communities associated with ambrosia beetles. No exclusion criteria were applied in the selection of articles for inclusion in this review. Identifying the key bacterial players among ambrosia beetle symbionts and elucidating their functional roles within this symbiotic system would greatly enhance our understanding of how fungal gardens are managed by these beetles and potentially open new scenarios for the management of harmful ambrosia beetle species.

## 2 Studies on the bacterial communities associated with ambrosia beetles and most common targeted species

A total of eight publications using metabarcoding to describe the bacterial communities associated with ambrosia beetles were identified during the literature review (Table 1). Most of these studies focused on ambrosia beetle species within the subfamily Scolytinae (Hulcr et al., 2012; Ibarra-Juarez et al., 2018, 2020; Nuotclà et al., 2021; Diehl et al., 2022, 2023; Calleros-González et al., 2024), whereas only one study examined the bacterial community of a species within the subfamily Platypodinae (Nones et al., 2021). Among the Scolytinae, most investigations focused on species in the tribe Xyleborini, whereas only one study included species from a different tribe (i.e., Cortylini) (Table 1). In total, 13 species have been studied so far. The most frequently targeted species were *Xyleborus affinis* and *Xyleborinus saxesenii* (i.e., in three articles), followed by *Xyleborus bispinatus* (i.e., in two articles). Conversely, *Corthylus consimilis*, *Dryocoetoides capucinus*, *Euwallacea discretus*, *Monarthrum dimidiatum*, *Platypus cylindrus*, *Xyleborus glabratus*, *Xyleborus volvulus*, *Xylosandrus crassiusculus*, *Xylosandrus germanus*, and *Xylosandrus morigerus* were targeted in only one study (Table 1).

**Table 1:** Metabarcoding studies investigating the bacteriome associated with ambrosia beetles.

References	Aims	Ambrosia beetle species	Subfamily	Tribe	Sample origin and collection	Primers (16S)	Sequencing method	Main findings
Hulcr et al. (2012)	To describe the microbial communities in the mycetangia of beetle species from different locations, looking for evidence of obligated symbionts	*Xyleborus affinis*	Scolytinae	Xyleborini	Different locations. Wild insects obtained by rearing naturally colonized logs or from traps baited with ethanol. Emerging females. Surface disinfected. Dissected mycetangia	515F/806R	454 Pyrosequencing	Certain phylotypes, including bacteria in Burkholderiales, *Mycoplasma*, and Pseudomonadales, are common inhabitants of the mycetangia, but it is unlikely that they act as obligated symbionts. Bacterial communities were more species-specific than locality-specific
		*Xyleborus bispinatus*						
		*Xyleborus glabratus*						
		*Xylosandrus crassiusculus*						
		*Xylosandrus germanus*						
Ibarra-Juarez et al. (2018)	To compare the microbial communities of wild and laboratory reared beetles	*Xyleborus affinis*	Scolytinae	Xyleborini	Different locations. Wild insects obtained by rearing naturally colonized logs or from traps baited with ethanol and insects reared in artificial media. Wild emerging females and mature insects from dissected reared media. Surface disinfected. Head + thorax and abdomen were processed separately.	341F/785R	Illumina	Bacterial communities were predominantly composed of the genera *Burkholderia, Erwinia, Sphingobacterium*, and *Stenotrophomonas*. Field-collected individuals exhibited greater microbial richness than their laboratory-reared counterparts, and the species of plants used in the sawdust for the rearing medium impacted the abundance of certain bacterial genera
		*Xyleborus bispinatus*						
		*Xyleborus volvulus*						
Ibarra-Juarez et al. (2020)	To describe how the microbial communities change over time both considering the different developmental stages and the fungal gardens. Additionally, functions of the communities are predicted, and microscopic description of the fungal gardens is performed.	*Xyleborus affinis*	Scolytinae	Xyleborini	Single location. Artificially reared insects. Eggs, larvae, pupae, mature and emerging adults. Head + thorax and abdomen were processed separately. Fungal gardens were also studied.	341F/785R	Illumina	Phylotypes of *Burkholderia, Enterobacter*, and *Stenotrophomonas* were dominant in all samples, suggesting that bacteria likely play significant roles in the fungal gardens, even prior to their full colonization by the mature cultivars of the nutritional fungal mutualist.
Nones et al. (2021)	To describe the microbial communities in the mycetangia of males and females. Additionally, microscopic description of the mycetangia is perfomed.	*Platypus cylindrus*	Platypodinae	Platypodini	Single location. Wild insects obtained by rearing naturally colonized logs. Mature insects from the dissected logs. Surface disinfected. Dissected mycentangia of males and females.	515F/806R	Illumina	Bacteria in the mycetangia exhibit sex-specific associations, with some bacteria being more abundant in either males or females, and the differences are linked to the different plant tissues that each sex colonize
Nuotclà et al. (2021)	To describe how the microbial communities of insects is affected by the moisture in the artificial media used for rearing. Additionally, behavior analyses of the insects is performed.	*Xyleborinus saxesenii*	Scolytinae	Xyleborini	Single location. Artificially reared insects. Whole insects, emerging females.	Hyb515F/Hyb806R	Illumina	While the composition of the mycobiota is altered by atypical moisture conditions during rearing, the bacterial community carried by the female dispersers remains significantly unaffected by the treatment
Diehl et al. (2022)	To compare the microbial communities in fungal gardens with adults vs. without adults.	*Xyleborinus saxesenii*	Scolytinae	Xyleborini	Single location. Artificially reared fungal gardens.	515F/806R	Illumina	Microbial community within the fungal gardens is influenced by the presence of the insect. The community was dominated by genera within the phylum Pseudomonadota, such as *Acinetobacter, Erwinia, Ochrobactrum, Pseudoxanthomonas,* and *Wolbachia* with *Microbacterium* from the phylum Actinomycetota also being relatively abundant. Gardens without beetles generally exhibited a richer diversity of bacteria
Diehl et al. (2023)	To describe how the microbial communities change over time in the fungal gardens.	*Xyleborinus saxesenii*	Scolytinae	Xyleborini	Single location. Wild and artificially reared fungal gardens	515F/806R	Illumina	Significant shifts in bacterial communities were not observed, but only specific ASVs of *Erwinia* and *Pseudoxanthomonas* were consistently present and highly abundant across all cultivars and through time
Calleros-González et al. (2024)	To describe the microbial communities of a number of ambrosia beetle species	*Corthylus consimilis*	Scolytinae	Corthylini	Single location. Wild insects obtained by rearing naturally colonized logs. Emerging insects. Surface desinfected. Head + thorax and abdomen were processed separately.	341F/785R	Illumina	*Wolbachia* was present exclusively in the head-thorax region of E. discretus. Bacterial communities clustered primarily by insect species. The most abundant bacterial genera were *Acinetobacter, Pseudomonas, Stenotrophomonas*, and *Sphingobacterium*
		*Monarthrum dimidiatum*						
		*Dryocoetoides capucinus*		Xyleborini				
		*Euwallacea discretus*						
		*Xylosandrus morigerus*						

The studies are listed in temporal order from the oldest to the newest. Aim, targeted ambrosia beetle species, methodology used to sample the beetles and to analyze the bacteriome, and main findings are reported for each study.

Among the eight studies that we summarized in this review, only two were specifically designed to investigate the bacterial community of targeted ambrosia beetles (i.e., Hulcr et al., 2012; Nones et al., 2021), whereas the others included data on fungal communities as well. Only one study examined specimens from multiple geographic locations (Hulcr et al., 2012). Most studies characterized the microbiota using the whole adult beetle, which limited insights both into the compartmentalization of bacterial communities on the beetle body and into possibly existing differences between bacterial communities of adults vs. preimaginal stages (Table 1). Some research has instead been conducted on the bacterial community present inside beetle mycetangia and inside the galleries (Table 1).

## 3 What is currently known on the bacterial community of ambrosia beetles

Studies carried out so far indicate that the bacterial communities associated with ambrosia beetles are species-specific. This pattern might be linked to the differences existing in the community of fungal mutualists associated to different ambrosia beetle species, which likely affect their associated bacterial community. For example, species of *Platypus, Xyleborinus*, and *Xyleborus* are mainly associated with fungi from the family Ophiostomataceae, *Euwallacea* species with fungi from the family Nectriaceae, and all other ambrosia beetle species considered in this review with fungi from the family Ceratocystiaceae (Johnson et al., 2018; Biedermann and Vega, 2020). These three fungal clades have independently evolved as mutualists of ambrosia beetles, and it is likely that their roles and functions within the symbiosis differ fundamentally (Johnson et al., 2018; Biedermann and Vega, 2020). Consequently, bacterial symbionts may also vary between these clades, fulfilling different ecological roles. Despite the highlighted differences among different ambrosia beetle species, certain microbial taxa tend to dominate across beetle species and even above-mentioned clades. For example, ambrosia beetle bodies are generally dominated by Gram-negative bacteria from the phyla Bacteroidota and Pseudomonadota, even though Gram-positive taxa such as Actinomycetota and Bacillota can also be prevalent (Hulcr et al., 2012; Ibarra-Juarez et al., 2018, 2020; Calleros-González et al., 2024). Fungal gardens cultivated by ambrosia beetles are instead mainly composed of Gram-negative bacteria in the phyla Pseudomonadota and Bacteroidota (Ibarra-Juarez et al., 2020; Nuotclà et al., 2021; Diehl et al., 2022, 2023). This may be due to the beetles’ active farming behavior and the influence of the plant substrate that shapes the microbiota within their nests. Notably, some bacterial taxa appear to be conserved across ambrosia beetles and their bark beetle ancestors. For instance, a clade of Erwiniaceae has been consistently detected in association with multiple species of Scolytinae (Cambronero-Heinrichs et al., 2023). This suggests that certain bacterial lineages may have long-standing associations with these insects, a pattern that may extend to other bacterial taxa as well.

The first study employing metabarcoding to characterize the bacterial communities associated with ambrosia beetles focused on the mycetangia of field-collected adult females of five Xyleborini species: *X. affinis*, *X. bispinatus*, *X. glabratus*, *X. crassiusculus*, and *X. germanus* (Hulcr et al., 2012). Prior to this study the presence of bacteria in mycetangia of ambrosia beetles had only been hypothesized (Grubbs et al., 2011). Hulcr et al. (2012) demonstrated that certain phylotypes, including Burkholderiales (Pseudomonadota), *Mycoplasma* (Mycoplasmota), and Pseudomonadales (Pseudomonadota), are common inhabitants of ambrosia beetle mycetangia. These phylotypes were detected in over half of the amplified samples and were consistently found across the different geographic regions where the analyzed individuals were collected. However, no universally present bacterial phylotype was found, which either indicates that there are no obligate bacterial symbionts, or more likely that they are present somewhere else in the beetle body. Hulcr et al. (2012) also found that the bacterial communities were more species-specific than locality-specific, supporting vertical transmission rather than environmental acquisition. Furthermore, the non-native ambrosia beetle species *X. glabratus*, *X. crassiusculus*, and *X. germanus* harbored unique bacterial communities which were distinct from those associated with ambrosia beetle species native to North America.

Nones et al. (2021) analyzed the bacterial community present in beetle mycetangia using field-collected specimens, specifically targeting *Platypus cylindrus* (Platypodinae), a significant pest of cork oak. Unlike Xyleborini beetles, both male and female of this platypodid species disperse and possess mycetangia, even though the latter are larger and more developed in females (Cassier et al., 1996). In *P. cylindrus*, males are the first to colonize suitable hosts. They first excavate initial galleries in the bark and in the phloem, then release aggregation pheromones to attract females. Females deepen the galleries started by the males reaching the xylem, where the next generation develops (Sousa and Inácio, 2005). Nones et al. (2021) showed that bacterial communities in the mycetangia of *P. cylindrus* are sex specific, with some groups being more abundant in either males or females, difference that might be linked to the different plant tissues colonized by each sex. The authors also suggested potential roles of the most abundant bacteria, such as the removal of plant tannins and polysaccharides (Nones et al., 2021), although these functions have yet to be experimentally verified.

Calleros-González et al. (2024) investigated the microbiota of five ambrosia beetle species, two Corthylini species (*C. consimilis* and *M. dimidiatum*), and three Xyleborini species (*D. capucinus*, *E. discretus*, and *X. morigerus*) (Table 1). In this study, the head, the thorax, and the abdomen of females were processed separately, revealing that the intracellular genus *Wolbachia* was present exclusively in the head-thorax region of *E. discretus*. Additionally, dissimilarity indices indicated that bacterial communities were species-specific, corroborating the findings by Hulcr et al. (2012). The most abundant genera associated with the five ambrosia beetle species examined were *Acinetobacter* (Pseudomonadota), *Pseudomonas* (Pseudomonadota), *Stenotrophomonas* (Pseudomonadota), and *Sphingobacterium* (Bacteroidota), which have been identified as highly abundant symbionts of other ambrosia beetle species (Hulcr et al., 2012; Ibarra-Juarez et al., 2018). Although studies describing the functional roles of these abundant bacteria in ambrosia beetles are lacking, *Pseudomonas* species are proposed to play nutritional and defensive roles in the closely related bark beetles (Saati-Santamaría et al., 2021) and might have the same role for ambrosia beetles hosting them.

Ibarra-Juarez et al. (2018) also compared the bacterial communities of adult Xyleborini beetles across multiple species, incorporating a comparison between wild and laboratory-reared insects. This study demonstrated that the bacterial communities of *X. affinis*, *X. bispinatus*, and *X. volvulus* were predominantly composed of the genera *Burkholderia* (Pseudomonadota), *Erwinia* (Pseudomonadota), *Sphingobacterium* (Bacteroidota), and *Stenotrophomonas* (Bacteroidota), and that rearing conditions significantly affected the structure of the bacterial communities. For instance, field-collected individuals exhibited greater microbial richness compared to their laboratory-reared counterparts, and the species of plants from which the sawdust used in the rearing medium was obtained impacted the abundance of certain bacterial genera. The impact of the host tree species on the bacterial community might occur for multiple reasons, including the effect of the chemical characteristics of wood tissue from different plant species and/or the presence of both dead and living DNA in the plant material used for rearing.

The remaining studies were based on specimens reared in laboratory conditions on artificial media prepared with host tree sawdust. Two model ambrosia beetle species were investigated in more details, i.e., *X. affinis* and *X. saxesenii.* Ibarra-Juarez et al. (2020) explored for the first time the temporal succession of bacteria across the life stages (i.e., eggs, larvae, pupae and adults) of *X. affinis* and in its fungal gardens within the galleries. Specific phylotypes of *Burkholderia* (Pseudomonadota), *Enterobacter* (Pseudomonadota), and *Stenotrophomonas* (Bacteroidota) were dominant in all samples. Authors indicated that these bacteria likely play a role also before the fungal garden is completely established on gallery walls, even though the exact functions were not assessed (Ibarra-Juarez et al., 2020). As shown for the same bacteria in other insect taxa (Santos et al., 2004; Dantur et al., 2015), these functions might be linked to plant polymer degradation and defensive roles.

**Figure 1:**
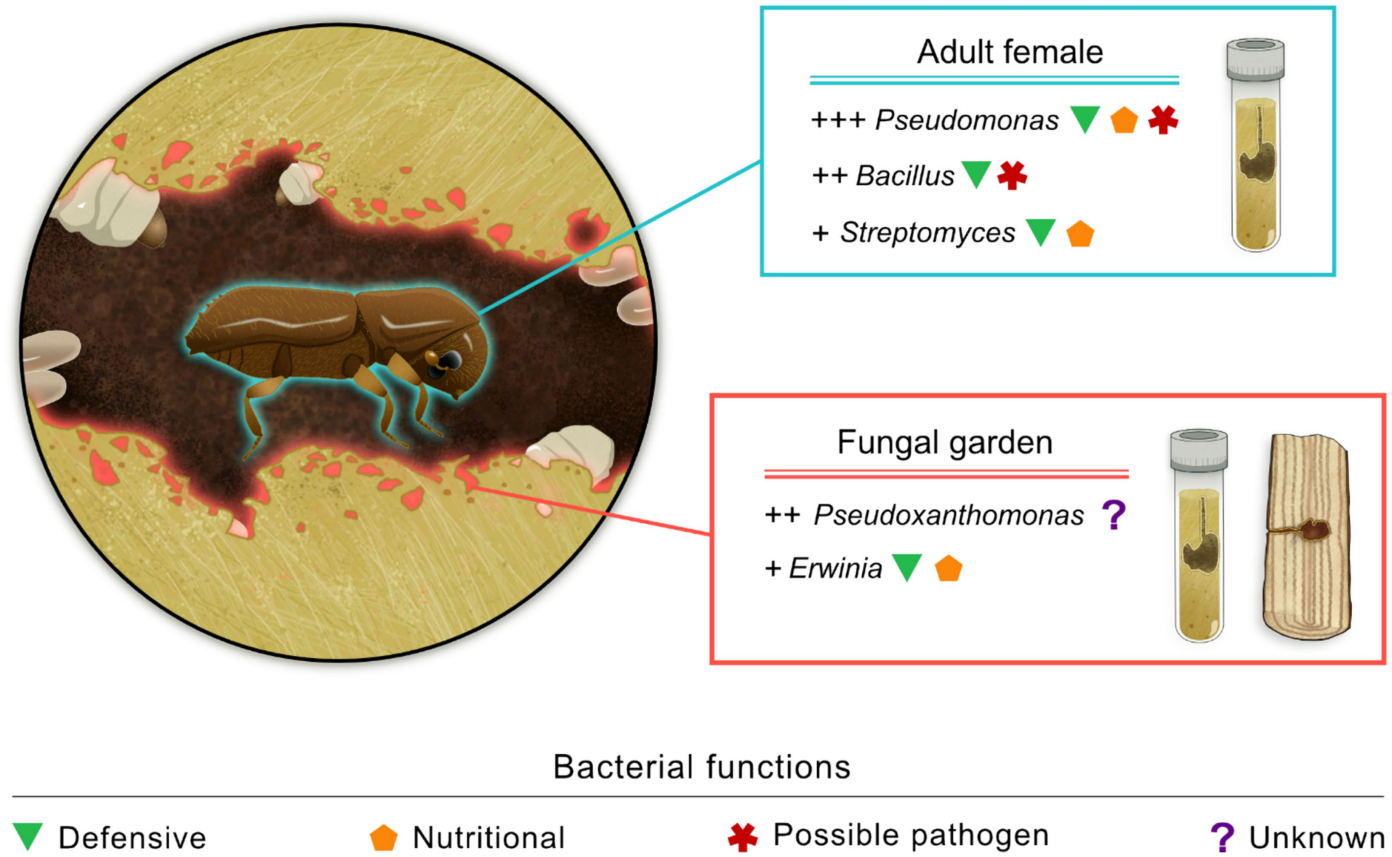
Overview of the bacterial communities associated with laboratory reared *Xyleborinus saxesenii* adult females and fungal gardens of *X. saxesenii* obtained from both rearing tubes and wood-samples. The relative abundance of bacterial genera is represented qualitatively using “+” symbols, and proposed functions are linked to the respective genera. Only the most abundant bacterial taxa are reported (image credit: L. Besana).

Considering *X. saxesenii*, Nuotclà et al. (2021) showed that the bacterial communities of emerging adult females from rearing tubes subjected to varying moisture levels were generally dominated by microbes in the genera *Bacillus* (Bacillota), *Pseudomonas* (Pseudomonadota), and *Streptomyces* (Actinomycetota) (Figure 1). Diehl et al. (2022) showed that the most abundant bacteria present in the fungal garden of lab-reared colonies of *X. saxesenii* belong to the genera in the phylum Pseudomonadota, such as *Acinetobacter, Erwinia, Pseudoxanthomonas*, *Ochrobactrum*, and *Wolbachia.* In the latter study, beetles were also experimentally removed from the rearing tubes in order to show the influence of the adult beetle presence on the microbial communities in the fungal gardens. The most abundant bacterial genera were not affected by the presence of the beetles in the gallery, even though fungal gardens without beetles generally exhibited a richer bacterial diversity than fungal gardens with the beetles. In another study, Diehl et al. (2023) compared the bacterial community in fungal gardens of field-collected and reared *X. saxesenii*, focusing on changes over time. Although significant shifts in bacterial communities were not observed, only specific Amplicon Sequence Variants (ASVs) of *Erwinia* and *Pseudoxanthomonas* were consistently present and highly abundant across all cultivars and through time, highlighting the importance of these phylotypes for *X. saxesenii*. The highly abundant phylotype of *Erwinia* was subsequently studied in greater detail, revealing that it possibly supports the ambrosia symbiosis by providing nutritional functions, such as synthesizing essential aminoacids and enzymes for the breakdown of plant polymers, and defending against competitors through antibiotic synthesis (Cambronero-Heinrichs et al., 2023). Among the other bacterial taxa found in association with *X. saxesenii* in the above-mentioned studies, only the defensive function of *Streptomyces* has been experimentally assessed in *X. saxesenii* (Grubbs et al., 2011). On the contrary, other functions of the same and other bacterial taxa have been described in bark beetles. For example, species of *Pseudomonas* and *Streptomyces* were shown to inhibit the growth of fungal competitors and produce enzymes capable of breaking down plant polymers (Grubbs et al., 2011; García-Fraile, 2018; Saati-Santamaría et al., 2021). Additionally, isolates of *Bacillus* and *Pseudomonas* were shown to detoxify plant defensive compounds, even though they may also have negative impacts on the insect (García-Fraile, 2018; Saati-Santamaría et al., 2021).

## 4 Molecular methods to study the bacterial communities of ambrosia beetles

Most studies on the bacterial communities associated to ambrosia beetles exploited 16S metabarcoding (Table 1). This method uses short DNA markers that are amplified and sequenced, providing information about the identity and abundance of the phylotypes in the microbial communities. For library preparation, studies carried out so far have employed two pairs of primers, or modified versions of them: 341F/785R and 515F/806R (Table 1). The 341F/785R primers target the V3-V4 region of the 16S rRNA gene and produce an amplicon with a maximum size of 446 bp (The Human Microbiome Project Consortium, 2012; Klindworth et al., 2013). In contrast, the 515F/806R primers, used first by the Earth Microbiome Project, target the V4 region and yield an amplicon with a maximum size of 315 bp (Gilbert et al., 2010; Caporaso et al., 2011). Comparative studies on the efficacy of these two primer pairs have demonstrated that the combination of 515F/806R recovers a higher number of ASVs or Operational Taxonomic Units (OTUs), allowing to amplify a higher diversity of bacterial taxa across different phylogenetic groups and thus providing a superior bacterial coverage (Eloe-Fadrosh et al., 2016; Lee et al., 2023). However, it is also now widely recognized that a single nucleotide modification of the 806R primer, changing a degenerate “H” (A, C, or T) to “N” (any base) to create the reverse primer 806RB (Apprill et al., 2015), significantly enhances metabarcoding results. The consensus today is that the optimal primer combination for characterizing the bacterial community of environmental samples is 515F/806RB (Eloe-Fadrosh et al., 2016; Lee et al., 2023). It is however important to highlight that, in the absence of internal and/or external standards, metabarcoding provides only semi-quantitative data of target sequences (Elbrecht and Leese, 2015; Vasselon et al., 2018). This is due to both methodological and biological factors that can lead to deviations from the actual bacterial abundance, such as sequence variation affecting primer binding efficiency, differences in amplification efficiency, target gene copy number variation, and ploidy (McLaren et al., 2019).

Other next-generation sequencing approaches could also offer valuable insights into the bacterial communities associated with ambrosia beetles. For instance, metagenomic sequencing, which targets the entirety of genomic material and not a limited number of markers, could provide more comprehensive information than metabarcoding. This approach can elucidate not only the identities of the phylotypes within the microbial community but also their potential functions in the ambrosia symbiosis and their possible interactions with host plants (Thomas et al., 2012). In addition, it can enable the identification of encoded metabolic pathways within microbial communities by detecting genes associated with key metabolic processes, and different tools can facilitate annotation of these pathways based on the presence of specific genes in the community (Kanehisa et al., 2016; Caspi et al., 2020). Furthermore, the reconstruction of metagenome-assembled genomes (MAGs) through binning techniques can allow for the assignment of species-specific functional potentials within the community (Alneberg et al., 2014; Kang et al., 2015). Although metagenomics provides valuable insights into the potential functions of microbial communities, actual functional activity must be confirmed through complementary techniques like meta-transcriptomics and meta-proteomics, which analyze gene expression and protein abundance, respectively (Franzosa et al., 2014). To date, only one metagenomic study is available for ambrosia beetles (Aylward et al., 2014). This study compared the microbial communities associated with *X. saxesenii* and other insects living in associations with nutritional fungal mutualists, including bark beetles, termites and leafcutter ants. Some bacterial phylotypes were shared across these insect taxa, suggesting that fungus-growing insects may have evolved convergent microbiotas that fulfill analogous functional roles (Aylward et al., 2014). On the other hand, meta-transcriptomic and meta-proteomic methods have never been applied to ambrosia beetles.

Genome sequencing of isolated microbes can also be highly valuable, particularly for investigating the functions of abundant and ecologically significant microorganisms. For example, the genomes of culturable bacteria which barcodes correspond to abundant or relevant ASVs/OTUs identified in metabarcoding studies have been targeted by Cambronero-Heinrichs et al. (2023). This approach facilitates studying the potential capacity of the annotated genes of microbes that may have beneficial roles for ambrosia beetles. It is cheaper than performing a metagenomic study and it also shows the benefit that obtaining isolates of relevant bacteria facilitates functional assays to experimentally test the roles of the identified genes (Cambronero-Heinrichs et al., 2023).

Other methods beyond sequencing can provide insights into other characteristics of the microbial communities. For instance, fluorescent *in situ* hybridization (FISH), which uses DNA or RNA probes to detect specific complementary sequences within a sample (Huber et al., 2018), has the capability to reveal the compartmentalization of microbes within ambrosia beetle body (e.g., mycetangia or gut) or their fungal gardens. In this context, rRNA-FISH is commonly preferred because the 16S rRNA gene provides essential phylogenetic resolution for the identification and classification of bacterial taxa (Amann et al., 1995; Pernthaler et al., 2001). Moreover, the high abundance of rRNA within microbial cells makes it an ideal target for FISH, as it significantly enhances the sensitivity and reliability of the method (Amann and Fuchs, 2008). This technique has been used extensively to determine the location of insect-associated microbial symbionts (Shi et al., 2010). Histological sections provide the highest resolution for microbial detection, but FISH can also be performed on whole mounts of entire specimens, provided they are sufficiently translucent (e.g., eggs, larvae, pupae), as well as on dissected organs or crushed preparations, although this may result in lower resolution depending on the sample size, composition, and clarity (Shi et al., 2010; Kliot and Ghanim, 2016). However, to date FISH has not been used to study the microbiota of ambrosia beetles.

It is also crucial to emphasize the importance of experimental approaches for testing hypotheses regarding factors that influence ambrosia beetle microbiota and the function of ambrosia beetle bacterial associates. In fact, there are only a few examples of similar targeting ambrosia beetles (Nuotclà et al., 2021; Diehl et al., 2022). Descriptive methods, such as metabarcoding for characterizing bacterial communities, are valuable for hypothesis generation but insufficient for validation unless linked to experimental data. In addition, taxonomic descriptions alone do not provide insight into the functional roles of microbes. Thus, molecular methods should not be employed merely for community description but should be integrated into experimental designs to rigorously test hypotheses regarding microbial function and factors affecting its composition.

## 5 Sampling methods to obtain ambrosia beetle individuals for studying their bacterial community

Two primary approaches for capturing adult individuals in the field have been employed so far in studies investigating ambrosia beetle microbiome: (i) ethanol-baited traps (Hulcr et al., 2012; Ibarra-Juarez et al., 2018) and (ii) collection either from galleries within trees or from emergence cages in which colonized cut logs were stored (Ibarra-Juarez et al., 2018; Nones et al., 2021; Diehl et al., 2023; Calleros-González et al., 2024; Table 1). An important consideration when using traps is whether the adult beetles are collected wet (i.e., trap or trap collector cup containing liquids) or dry (i.e., trap or trap collector cup containing no liquids). The presence of liquid in the collector cup should be avoided, as this could lead to cross-contamination among trapped specimens. Instead, crumpled paper humidified with sterile water and placed in the collector cup might reduce eventual cross-contamination processes keeping the beetles alive for a few days. Ensuring that the beetles do not die in the traps is also essential not only when subsequent rearing is planned but also because the microbiota begins to shift in deceased insects, with saprophytic microbes rapidly dominating the communities and affecting results of metabarcoding studies (Preiswerk et al., 2018).

Sampling adult beetles from their galleries or using those emerged from infested trees can be considered a better approach than trapping when the aim is to study microbial communities. In fact, this approach allows to further reduce the likelihood of cross-contamination among individuals. Additionally, since it is known that the host tree can influence ambrosia beetle mycobiome (Morales-Rodríguez et al., 2021), obtaining adults from a given tree species allows to avoid the confounding effect of this variable when analyzing their associated bacterial communities. On the other hand, the dissection of colonized woody material might be time-consuming, especially considering that visible external symptoms (e.g., entry holes) are not always an indication of fully-developed and active galleries within the wood (Cambronero-Heinrichs et al., 2024). Furthermore, the microbiota of ambrosia beetles and their fungal cultivars undergoes a succession process, with community composition varying according to the developmental stage of the beetles (Ibarra-Juarez et al., 2018; Diehl et al., 2023). For example, the microbiota differs between fully developed adults within the tunnels and newly emerging females (possibly due to non-active vs. active mycetangial glands; Ibarra-Juarez et al., 2020). This variation must be considered when designing a study and it is very difficult to control when using individuals from naturally infested trees.

Using beetles reared on artificial media in lab-conditions is another potential approach to obtain individuals for subsequent analysis. However, it must be kept in mind that the microbiota of lab-reared beetles strongly differs from that of their wild counterparts (Ibarra-Juarez et al., 2018; Diehl et al., 2023), and that some essential organisms may get lost under lab conditions. On the other hand, laboratory rearing allows for experimental manipulations (e.g., antibiotic treatments to exclude specific taxa) and the control of multiple variables, such as the plant species used in the rearing medium (Ibarra-Juarez et al., 2018) and the addition of nutrients, chemicals or microbes. It also enables the collection of specific developmental stages for detailed examination of both the beetles and their fungal gardens (Ibarra-Juarez et al., 2020; Diehl et al., 2023).

## 6 Conclusion

Although molecular techniques have advanced the study of the bacterial communities associated with ambrosia beetles, this topic remains largely unexplored. The main knowledge gap lies in the understanding of the functional roles of the dominant bacteria. Analyses based on metabarcoding studies have relied so far on the assumption that the most abundant bacteria might have the same roles they play in other insects, such as the closely related bark beetles. Nonetheless, this approach may lead to misinterpretations. While describing the microbial community of ambrosia beetles is an essential first step, more studies should aim to use other techniques such as (meta-) genomics, transcriptomics and bioassays to assess functional roles. Additionally, studies should be guided by experimental design to test hypotheses regarding the various factors influencing microbial composition and function. It would also be important to avoid generalizations and not to assume that functions found in a certain species are also transferable to other species. This applies in particular to groups of ambrosia beetles that are associated with independently evolved lineages of fungi. For bacteria, it is important to note that phylogenetically different taxa may perform similar roles for their hosts, and even if bacterial communities differ taxonomically, their functional roles may remain similar. Besides the dominant bacteria, future studies should also target the less abundant phylotypes as they may also play significant roles that are currently unexplored. In symbiosis, abundances of specific microbial taxa are not a good proxy for the importance to their hosts, especially if these are semi-quantitative and are based on metabarcoding. Moreover, it would be crucial to better investigate the compartmentalization of bacterial communities within ambrosia beetle bodies, differences with preimaginal developmental stages, and their fungal gardens. For instance, no studies have specifically focused on the gut microbiota of ambrosia beetles, and it remains unknown whether certain abundant bacteria form biofilms or are localized within specific organs. Finally, the interaction between beetles, their microbiota, and their host plants is largely overlooked. Little is known about the influence of plant host defenses on the bacterial communities associated with ambrosia beetles or whether the beetles can acquire and transmit bacteria from these hosts. It is possible that some of the bacterial symbionts not only play a role for the insect but also for the plants. For example, certain dominant bacteria within the microbiota of ambrosia beetles may express virulence factors, potentially harming the trees and helping the beetles in colonization.
